# Death of Dr. Daniel Drake

**Published:** 1852-12

**Authors:** 


The distinguished and venerable Dr. DANIEL DRAKE, who has so long filled a prominent part in the profession of the West, died at Cincinnati during the last month. We shall present a more detailed notice of his rise and career hereafter.

